# Bifunctional Oxygen Electrocatalysis on Mixed Metal Phthalocyanine-Modified Carbon Nanotubes Prepared via Pyrolysis

**DOI:** 10.1021/acsami.1c06737

**Published:** 2021-08-24

**Authors:** Yogesh Kumar, Elo Kibena-Põldsepp, Jekaterina Kozlova, Mihkel Rähn, Alexey Treshchalov, Arvo Kikas, Vambola Kisand, Jaan Aruväli, Aile Tamm, John C. Douglin, Scott J. Folkman, Ilario Gelmetti, Felipe A. Garcés-Pineda, José Ramón Galán-Mascarós, Dario R. Dekel, Kaido Tammeveski

**Affiliations:** † Institute of Chemistry, University of Tartu, Ravila 14a, 50411 Tartu, Estonia; ‡ Institute of Physics, University of Tartu, W. Ostwald Street 1, 50411 Tartu, Estonia; § Institute of Ecology and Earth Sciences, University of Tartu, Vanemuise 46, 51014 Tartu, Estonia; ∥ The Wolfson Department of Chemical Engineering, Technion—Israel Institute of Technology, 3200003 Haifa, Israel; ⊥ Institute of Chemical Research of Catalonia (ICIQ), The Barcelona Institute of Science and Technology (BIST), 43007 Tarragona, Spain; # Catalan Institution for Research and Advanced Studies (ICREA), Passeig Llüis Companys 23, 08010 Barcelona, Spain; ∇ The Nancy & Stephen Grand Technion Energy Program (GTEP), Technion—Israel Institute of Technology, 3200003 Haifa, Israel

**Keywords:** oxygen reduction reaction, oxygen evolution reaction, metal phthalocyanines, carbon nanotubes, electrocatalysis, anion-exchange membrane fuel cell

## Abstract

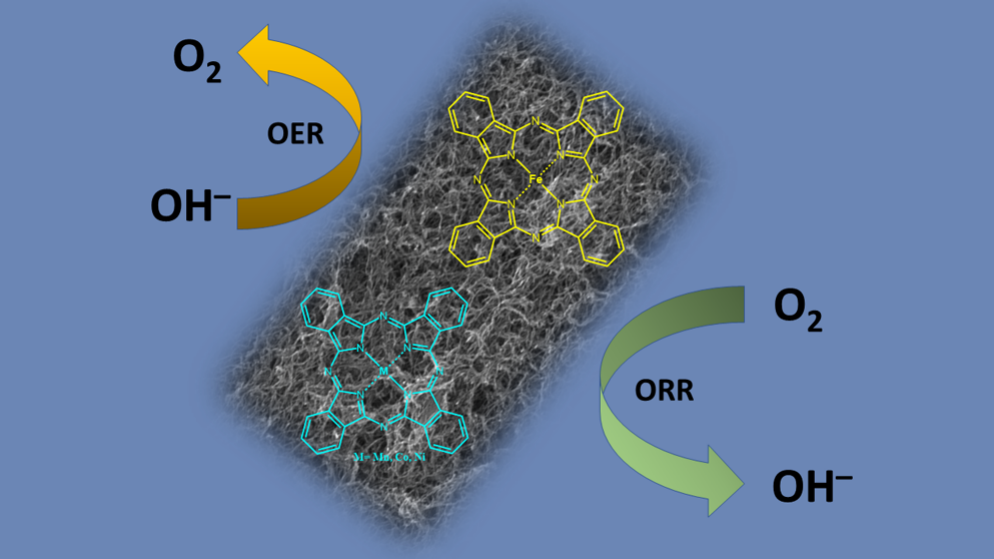

Non-precious-metal catalysts are promising alternatives for Pt-based cathode materials in low-temperature fuel cells, which is of great environmental importance. Here, we have investigated the bifunctional electrocatalytic activity toward the oxygen reduction reaction (ORR) and the oxygen evolution reaction (OER) of mixed metal (FeNi; FeMn; FeCo) phthalocyanine-modified multiwalled carbon nanotubes (MWCNTs) prepared by a simple pyrolysis method. Among the bimetallic catalysts containing nitrogen derived from corresponding metal phthalocyanines, we report the excellent ORR activity of FeCoN-MWCNT and FeMnN-MWCNT catalysts with the ORR onset potential of 0.93 V and FeNiN-MWCNT catalyst for the OER having *E*_OER_ = 1.58 V at 10 mA cm^–2^. The surface morphology, structure, and elemental composition of the prepared catalysts were examined with scanning electron microscopy, X-ray diffraction, and X-ray photoelectron spectroscopy. The FeCoN-MWCNT and FeMnN-MWCNT catalysts were prepared as cathodes and tested in anion-exchange membrane fuel cells (AEMFCs). Both catalysts displayed remarkable AEMFC performance with a peak power density as high as 692 mW cm^–2^ for FeCoN-MWCNT.

## 1 Introduction

With the increasing demand for energy and the limited amount of fossil fuels, the development of the electric automobile and the generation of renewable energy are of great interest toward a sustainable world. Alternative clean energy technologies, such as fuel cells, metal-air batteries, and water electrolyzers have gained much attention for the sustainable development of human society.^1^ For example, metal-air batteries are considered to be an excellent choice as energy storage devices to buffer the unpredictable demand of energy due to their high theoretical energy density and low production price. These batteries can be used in various applications ranging from very small button cells for hearing aids to very large batteries for electric vehicles and large-scale energy storage.^2,3^ Two electrochemical reactions take place at air electrode of rechargeable zinc-air batteries, i.e., the oxygen reduction reaction (ORR) and the oxygen evolution reaction (OER). However, due to the multielectron transfer routes and slow kinetics, the ORR and OER generally require high overpotential.^4,5^ It is critical to prepare a reversible and efficient oxygen electrode (cathode) for practical application. Platinum-based nanomaterials have been spotted as the most efficient electrocatalyst for the ORR.^6,7^ For the OER, noble metals such as ruthenium or iridium and their oxides have shown state-of-the-art OER activity.^8^ However, their high cost and mediocre stability hinder their use in commercial applications.^6^ Thus, substantial attempts have been made toward the development of high-performance non-precious-metal electrocatalysts for the ORR and OER.^9−20^

It has been experimentally and theoretically established that the transition-metal and nitrogen-doped carbon materials (M-N-C) are promising candidates for the ORR and OER due to their earth-abundant content, low price, and promising electrocatalytic properties.^11,21−23^ In the past decade, extensive work has been carried out to prepare M-N-C electrocatalysts, e.g., by pyrolysis of nitrogen-containing compounds, carbon sources, and transition-metal salts.^24−26^ Nonetheless, most of them still show inferior ORR performance compared to Pt-based catalysts, primarily because of the lower surface density of catalytically active sites and sluggish ORR kinetics.^27^ It has been well established that the incorporation of M-N_x_ moieties within conductive carbon matrices (e.g., via metal porphyrins and phthalocyanines) leads to M-N*_x_*-containing electrocatalysts for the ORR.^26,28−36^ For example, iron phthalocyanine (FePc) materials have been treated by high-temperature pyrolysis along with carbide-derived carbon for the preparation of an Fe-N_4_ complex, which exhibits improved ORR activity.^29^ Cheng and co-workers prepared bifunctional electrocatalysts for the ORR and OER by atomically dispersed FePc sites anchoring on defective carbon nanosheets.^37^ Many transition-metal phthalocyanine-based materials have been prepared for the improvement of oxygen-involving reactions (ORR and OER).^38−43^ However, there are only a few reports of bimetallic phthalocyanine materials for oxygen electrocatalysis, and often, complex and time-consuming synthesis procedures have been employed to prepare bifunctional ORR/OER catalysts.^44,45^

Transition-metal macrocycle-derived M-N-C materials show better activity in alkaline than acidic medium, which means that these materials can be used in low-temperature anion-exchange membrane fuel cells (AEMFCs).^46^ Due to the fact that these cells allow the application of non-precious-metal catalyst materials, AEMFCs have many other benefits over proton-exchange membrane fuel cells (PEMFCs), including but not limited to a wide choice of fuels and faster ORR kinetics.^47−49^ The main drawback for AEMFCs was poor conductivity of the anion-exchange membranes (AEMs); however, in recent studies, there are reports on AEMs exhibiting high hydroxide conductivity,^50−55^ sometimes exceeding 200 mS cm^–1^, significantly higher than the proton-conducting Nafion-based membranes.^56,57^ With the development of highly conducting AEMs, we need suitable catalysts for the enhancement of AEMFC performance. M-N-C-type catalysts are promising candidates due to their high ORR electrocatalytic activity, resistance toward corrosion, and high active surface area.^25,58,59^

In this work, we developed Fe-containing bimetallic—FeMn, FeNi, and FeCo—phthalocyanine complexes on multiwalled carbon nanotubes (MWCNT), forming M-N-C-type electrocatalysts for oxygen-involving reactions. The Fe-containing bimetallic catalysts were conveniently obtained by the one-step pyrolysis process of mixing different metal phthalocyanines and MWCNTs to enhance the electronic conductivity. The ORR and OER activities of the prepared catalysts were evaluated in alkaline media using the rotating disk electrode (RDE) method. The most active catalysts were also tested in AEMFC as well as anion-exchange membrane electrolyzer (AEMEL) configurations as cathode catalysts.

## 2 Experimental Section

### 2.1 Reagents

Iron phthalocyanine (FePc, 90%, Acros Organics), manganese phthalocyanine (MnPc, Alfa Aesar), cobalt phthalocyanine (CoPc, Alfa Aesar), nickel phthalocyanine (NiPc, 95%, Alfa Aesar), and multiwalled carbon nanotubes (MWCNTs, Nanocyl S.A., Belgium) were used as obtained. Potassium hydroxide (85%, Sigma-Aldrich) was used as the electrolyte in Milli-Q water, ethanol was used for mixing the metal phthalocyanines (MPc), and MWCNTs and 5 wt % Nafion (Aldrich) were used for the preparation of the catalyst inks in 2-propanol. Pt/C (20%, E-TEK, Inc.) and RuO_2_ (99.9%, Alfa Aesar) were employed for comparison purposes in the case of ORR and OER measurements, respectively. All glassware was cleaned thoroughly before experiments.

### 2.2 Synthesis of FeMnN-MWCNT, FeCoN-MWCNT, and FeNiN-MWCNT

The FeMnN-MWCNT catalyst was prepared by mixing 20 mg of MWCNTs, 10 mg of FePc, and 10 mg of MnPc in 5 mL of ethanol. The mixture was sonicated for 1 h to obtain a homogeneous suspension. Ethanol was evaporated in an oven at 60 °C. Then, the mixture was pyrolyzed at 800 °C for 1 h in N_2_ atmosphere. Other catalysts (FeCoN-MWCNT, FeNiN-MWCNT) were also prepared by the same method, but instead of using MnPc, CoPc for FeCoN-MWCNT and NiPc for FeNiN-MWCNT were employed. Single metal phthalocyanine-modified MWCNT catalysts were also synthesized for comparison purposes (see Table 1).

**Table 1:** Preparation of Single and Bimetallic and Nitrogen-Containing MWCNT-Based Electrocatalysts Using Different Metal Phthalocyanines

catalyst	FePc (mg)	MnPc (mg)	CoPc (mg)	NiPc (mg)	MWCNT (mg)
FeMnN-MWCNT	10	10			20
FeCoN-MWCNT	10		10		20
FeNiN-MWCNT	10			10	20
FeN-MWCNT	10				20
MnN-MWCNT		10			20
CoN-MWCNT			10		20
NiN-MWCNT				10	20

### 2.3 Characterization of the Catalysts

The surface morphology of the prepared catalysts was studied by scanning electron microscopy (SEM), and the images were taken using a high-resolution SEM, Helios Nanolab 600 electron-ion dual-beam microscope (FEI company) with 15 keV. For SEM measurements, the catalyst materials were sonicated in 2-propanol and Milli-Q water (1:1), followed by coating the suspension onto a polished glassy carbon (GC) disk and dried at 60 °C in an oven. The bulk content of the elements was determined by the SEM equipped with energy-dispersive X-ray analysis (EDX). Transmission electron microscopy analysis was performed in the scanning mode (STEM) at 200 kV using a Cs-probe-corrected transmission electron microscope (FEI Titan Themis 200). The crystallinity of the catalysts was determined by X-ray diffraction (XRD). The XRD analysis was performed by a Bruker D8 Advance diffractometer with Ni-filtered Cu Kα (λ = 1.54 Å) as a radiation source and a LynxEye line detector in the 2θ range of 5–90°. Micro-Raman spectra were recorded in the back-scattering geometry on an inVia Renishaw spectrometer together with an Olympus confocal microscope (50× objective) and an argon-ion laser operated at 514.5 nm. All samples were suspended in 2-propanol/Milli-Q water (1:1) and drop-coated on silicon substrates. To avoid thermal decomposition of the sample, the laser power density was minimized by decreasing the laser power and defocusing laser spot to about 15 μm. X-ray photoelectron spectroscopy (XPS) data were acquired using the Mg Kα X-rays (1253.6 eV) from a nonmonochromatic twin-anode X-ray tube (Thermo Scientific XR3E2) and an electron energy analyzer Scienta SES 100 (pass energy 200 eV). The survey scans were performed using a step size of 0.5 eV with a step duration of 0.2 s and averaged over five scans. For the high-resolution XPS spectra, a step size of 0.2 eV with a step duration of 0.2 s and 30 scans were averaged. The atomic concentration calculation and data analysis were performed with Casa XPS software using Gauss–Lorentz hybrid function (GL 70, Gauss 30%, Lorentz 70%). For the XPS analysis, catalyst materials were dispersed in 2-propanol/Milli-Q water (1:1); then, the mixture was drop-cast onto polished GC plates and dried at 60 °C.

### 2.4 Electrochemical Measurements

The electrocatalytic activity of the prepared materials was evaluated in a three-electrode cell configuration with a carbon rod as the auxiliary electrode, saturated calomel electrode (SCE) as the reference electrode, and a catalyst-coated GC rotating disk electrode (geometric area = 0.196 cm^2^) as the working electrode. A potentiostat/galvanostat PGSTAT30 (Metrohm-Autolab, The Netherlands) and NOVA software were used for the electrochemical analysis. For the RDE experiments, a CTV101 speed controller equipped with an EDI101 rotator (Radiometer) was used. For the preparation of catalyst suspension, 4 mg of catalyst was homogeneously dispersed by sonication for 1 h in a solution consisting of 790 μL of 2-propanol, 195 μL of Milli-Q water, and 15 μL of 5 wt % Nafion solution. After that, 10 μL of the catalyst suspension were drop-casted on a GC electrode to obtain 0.2 mg cm^–2^ catalyst loading and dried at 60 °C. The RDE measurements were performed in O_2_-saturated 0.1 M KOH electrolyte solution. Linear sweep voltammetry (LSV) data were recorded at different rotation rates (ω) of 360, 610, 960, 1900, and 3100 rpm at a scan rate (*ν*) of 10 mV s^–1^. The background current was measured in Ar-saturated 0.1 M KOH solution at 10 mV s^–1^ and subtracted from the experimental O_2_ reduction current to eliminate nonfaradic currents. The OER measurements were conducted in Ar-saturated 0.1 M KOH at 10 mV s^–1^. The electrochemical results reported in this work were iR-corrected, and all potentials were converted against the reversible hydrogen electrode (RHE) by the equation *E*_RHE_ = *E*_SCE_ + 0.241 V + 0.059 V × pH. The RDE experimental data were analyzed by the Koutecky–Levich (K–L) equation.^60^ The number of electrons (*n*) was calculated by the following equations
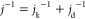
1where *j*, *j*_k_, and *j*_d_ represent measured, kinetic-limited, and diffusion-limited current densities, respectively.
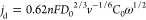
2where *n* is the electron transfer number per O_2_ molecule, *F* is the Faraday constant (96 485 C mol^–1^), *D*_0_ is the O_2_ diffusion coefficient (1.9 × 10^–5^ cm^2^ s^–1^), *v* is the kinematic viscosity of the electrolyte solution (0.01 cm^2^ s^–1^ for 0.1 M KOH), *C*_0_ is the concentration of O_2_ in bulk (1.2 × 10^–6^ mol cm^–3^), and *ω* is the rotation rate of the electrode (rad s^–1^).

### 2.5 AEMFC Testing

The gas diffusion electrode (GDE) method was used to make both anode (ACL) and cathode catalyst layers (CCL) for AEMFC testing, following previously published procedures.^28,54^ Before adding the catalyst layers to the gas diffusion layers, a thin 17 μm microporous layer of carbon was deposited to help elucidate flooding in the higher-current-density, mass transport region of the fuel cells.^48,61^ The PtRu loading for all anodes was 0.7 ± 0.03 mg_PtRu_ cm^–2^, resulting in an ACL of 69 μm thickness, while the M-N-C cathodes were loaded to 0.745 mg cm^–2^ with CCLs of 38 μm thickness. All electrodes, together with two 9 cm^2^ pieces of 5 μm thick FAA-3-05-rf anion-exchange membrane based on an FAA-3 polymer reinforced with porous ePTFE-film, delivered in HCO_3_^–^ form by Fumatech (Germany), were separately immersed in Petri dishes of 1 M KOH aqueous solution for 1 h. The solution in each Petri dish was changed every 20 min, to ensure full conversion of the AEM into its hydroxide form. Two AEMFCs were assembled between pairs of 5 cm^2^ single-serpentine graphite bipolar flow fields with PTFE gaskets, to arrive at a compression of 25% and torqued to 4.5 N m. The cells were tested in an 850E Scribner Associates Fuel Cell test station under H_2_–O_2_, followed by H_2_-air (CO_2_-free) flowing to the anode and cathode, respectively. All of the tests were performed at a cell temperature of 60 °C with similar operating conditions to compare the catalyst as unbiasedly as possible.

### 2.6 AEMEL Testing

Membrane electrode assemblies were fabricated using catalyst inks sprayed directly onto a Fumasep VM-FAA-3-10-rf 10 μm thick anion-exchange membrane reinforced with ePTFE from Fumatech (Germany). Catalyst inks were prepared using 70 mg of catalyst: FeNiN-MWCNT or RuO_2_ (99.9%, Alfa Aesar). The catalyst powder was mixed with 1.7 mL of ionomer solution (activated FAA-3 dissolved in ethanol 5% w:w, resulting in 50% w/w over catalyst powder) and ground in a mortar and pestle for 5 min. The black mixture was then placed in a 10 mL screw cap vial with 6 mL of a solution that was 3:1 isopropanol/water and sonicated for 30 min. The ink was then spray-deposited directly onto a preweighed, unactivated membrane that was taped to a Petri dish with a mask to expose a 32 mm diameter circle and heated to 80 °C. After the catalyst layer was deposited, the membrane was cooled to room temperature and weighed again to determine the catalyst loading (between 1.8 and 1.9 mg cm^–2^ for both catalysts). The membranes containing the OER catalysts were then hot-pressed at 120 °C under 1 ton of force applied using a hydraulic press for 5 min. After cooling to room temperature, they were gently removed from the hot press and oriented catalyst side down onto a titanium GDL. For the cathodic hydrogen evolution reaction (HER) catalyst, platinum on carbon GDL (0.5 mg cm^–2^ Pt, 325 μm thickness, purchased from Baltic FuelCells) was placed with the platinum side facing the membrane and Ti GDLs were placed in contact with the carbon GDL. The membrane electrode assemblies were encased in a Teflon cell with Monel pistons pressurized to 10 bar. The VM-FAA-3-10-rf membranes were activated by flowing 0.1 M KOH heated to 60 °C through the cell for 1 h prior to electrochemical experiments.

The performance of the FeNiN-MWCNT- and benchmark RuO_2_-based membrane electrode assemblies were evaluated in AEM electrolysis at 60 °C using 0.1 M KOH. The series resistance of all of the membrane electrode assemblies was ∼0.6 Ω·cm^2^ as determined by EIS. The LSV of the membrane electrode assemblies with the FeNiN-MWCNT catalyst, and RuO_2_ for comparison, was conducted at a scan rate of 1 mV s^–1^ from the open-circuit potential to a cell voltage of 2 V. Significant bubbling was observed from both the anodic and cathodic compartments at potentials greater than 1.5 V. To probe the stability of the FeNiN-MWCNT catalyst, chronoamperometry was conducted at 1.8 V for 2 h.

## 3 Results and Discussion

### 3.1 Morphology and Structural Characterization of the Catalysts

**Figure 1:**
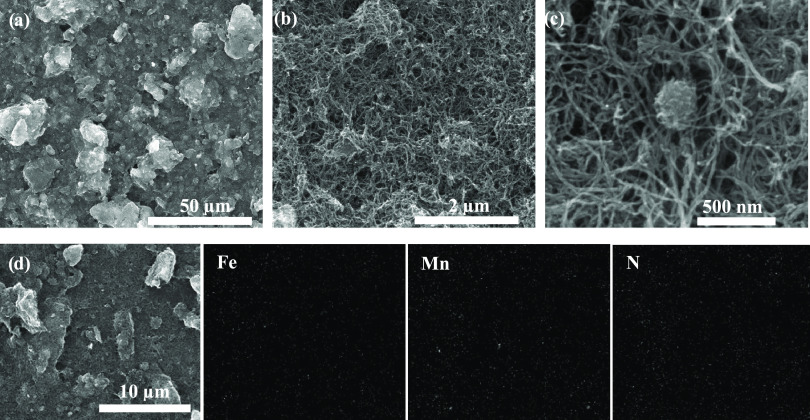
(a–c) SEM images and (d) SEM-EDX analysis with elemental mapping of FeMnN-MWCNT.

The microstructure and morphology of the prepared catalysts (FeMnN-MWCNT, FeCoN-MWCNT, and FeNiN-MWCNT) were investigated by SEM. The SEM images (Figures 1a–c and S1) indicate that all the prepared catalysts are composed of a similar structure of carbon nanotubes. The SEM images with higher magnification (Figure 1c) suggest that the carbon nanotubes are arranged in different directions, and some nanotubes agglomerated where there are some metal nanoparticles. This is also confirmed by the elemental analysis. For example, the EDX images suggest that all of the elements (Fe, Mn, N) are homogeneously distributed on carbon nanotubes in the case of FeMnN-MWCNT (Figures 1d and S2). Further, the elemental composition of the catalysts was evaluated, and results were presented in Table S1. The total content of metals found by EDX analysis corresponds to nearly 5 wt %, as expected from the theoretical calculation. It should be noted that the prepared bimetallic catalysts do not show any difference in surface morphology, which means that different metal phthalocyanines do not affect the morphology of the catalyst materials to a noticeable degree. Furthermore, STEM was also carried out to get some further insight into the prepared catalysts (see Figure S3). The STEM images showed that the metal is present in the form of nanoparticles, shown microscopically as knots/bundles. Upon closer inspection, fringes can be seen around the nanoparticles, which proves the presence of metal nanoparticles within carbon matrix, further validating the SEM results.

**Figure 2:**
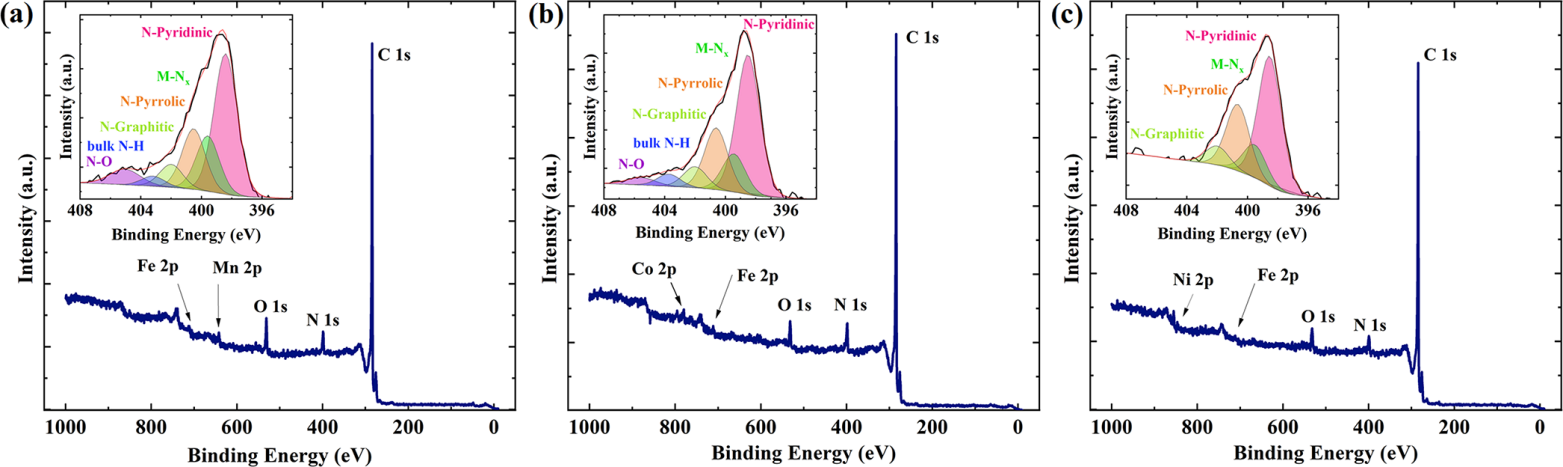
XPS survey spectra and N 1s high-resolution XPS spectra (inset) of (a) FeMnN-MWCNT, (b) FeCoN-MWCNT, and (c) FeNiN-MWCNT.

The surface elemental compositions of FeMnN-MWCNT, FeCoN-MWCNT, and FeNiN-MWCNT catalysts were investigated by XPS, and the results are presented in Figure 2 and Table 2. The XPS analysis indicates that there are five elements present on the surface of bimetallic catalysts, including C, N, O, and corresponding metals. From the N 1s region of high-resolution XPS spectra, N-graphitic, N–O, bulk N–H, and metal-coordinated nitrogen (M-N*_x_*) are observed, except in the case of FeNiN-CNT, which does not contain any N–O and bulk N–H type nitrogen (Figure 2 and Table S2). In all cases of the bimetallic catalysts, N-pyridinic type is in the highest content, followed by N-pyrrolic and then M-N*_x_* sites. N-pyridinic and M-N*_x_* are considered the most active sites for the ORR.^62,63^ The XPS analysis suggests that doping with MPc was successful as there are M-N*_x_* moieties present in all of the catalysts.

**Table 2:** Surface Elemental Composition of Catalysts as Determined by the XPS Analysis (atomic %)

element	FeMnN-MWCNT	FeCoN-MWCNT	FeNiN-MWCNT
C	88.7	89.2	92
N	4.9	6.0	3.5
O	5.6	4.0	3.9
Fe	0.2	0.2	0.2
Mn/Co/Ni	0.7	0.6	0.5

**Figure 3:**
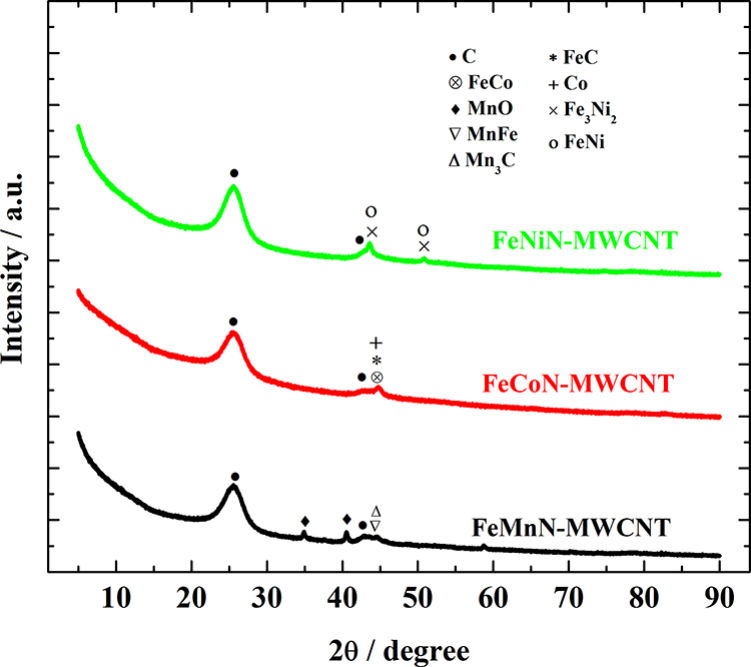
XRD patterns of the as-synthesized catalysts.

Further XRD analysis was used to study the crystallographic structure of the prepared catalysts. Figure 3 shows the XRD patterns of various Fe-based catalysts. The largest peak positioned at ∼25.6° is referred to the (002) plane of graphitic carbon. The other diffraction peaks around 40–50° demonstrate the presence of graphite (also visible in STEM images Figure S3c), metal carbide, metal oxide, and metal alloys (Figure 3).^62,63^ Furthermore, Raman spectra were recorded for pristine and as-synthesized catalysts (Figure S4). Compared to the unmodified MWCNTs, pyrolysis of mixed metal phthalocyanines in combination with MWCNTs leads to a strong broadening of the D and G bands due to the formation of defects introduced in carbon matrix by transition-metal and heteroatom doping (as was confirmed by the XPS analysis), thereby distorting the carbon nanotube structure.

### 3.2 Electrochemical Characterization of the Catalysts

**Figure 4:**
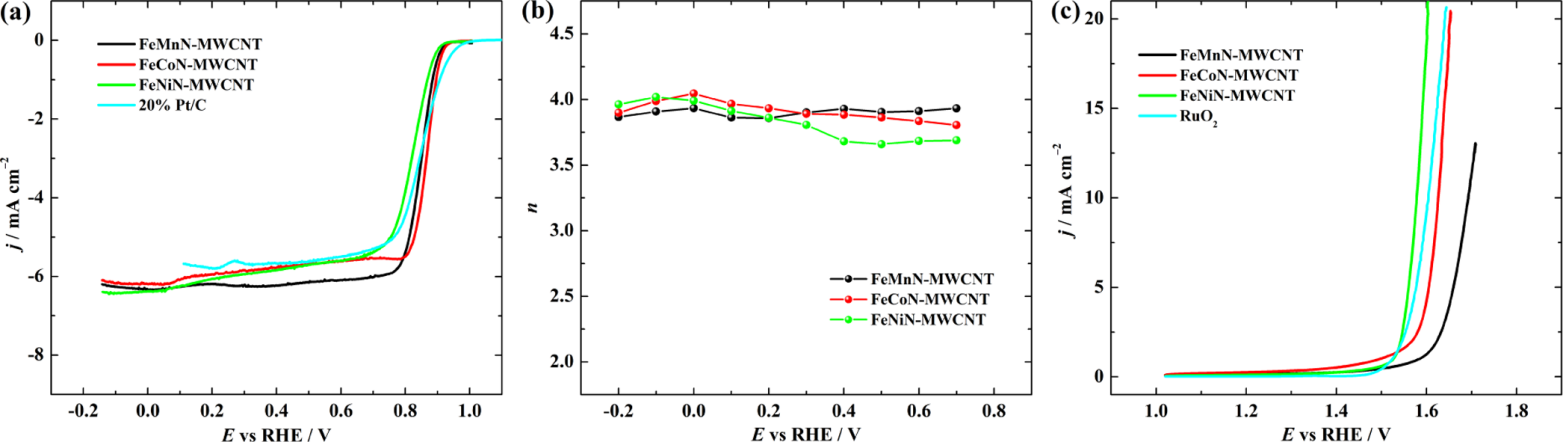
(a) ORR polarization curves recorded in O_2_-saturated 0.1 M KOH (*ν* = 10 mV s^–1^) at 1900 rpm; (b) *n* value as a function of potential; and (c) OER polarization curves recorded in Ar-saturated 0.1 M KOH electrolyte (*ν* = 10 mV s^–1^) for prepared catalysts.

The ORR activity of the catalysts was studied in O_2_-saturated 0.1 M KOH electrolyte. For comparison, the ORR performance of 20% Pt/C catalyst was also evaluated under similar conditions. First, cyclic voltammograms were measured in O_2_- and Ar-saturated electrolytes (Figure S5). As shown in Figure S5, all of the electrocatalysts show well-defined ORR cathodic peaks around 0.8 V in O_2_-saturated 0.1 M KOH solution, but in Ar-saturated solution, no peak appeared. The oxidation peak between 0.2 and 0.4 V has been observed and may refer to the formation of Fe(OH)_2_.^64^ The ORR performance of the electrocatalysts was further studied by the RDE method (Figure 4a). Figure S6 shows the RDE polarization curves of various catalysts recorded at different rotation rates. The FeCoN-MWCNT exhibits the highest ORR activity among all of the mixed metal phthalocyanine-modified MWCNT catalysts in terms of onset potential (*E*_onset_, potential at which the ORR current density reaches –0.1 mA cm^–2^) and half-wave potential (*E*_1/2_). Namely, FeCoN-MWCNT exhibits an onset potential of 0.93 V, which is 50 mV less than that of 20% Pt/C catalyst (0.98 V). The *E*_onset_ values for FeMnN-MWCNT and FeNiN-MWCNT are 0.93 and 0.92 V, respectively (Table 3). More importantly, the half-wave potential for the ORR on FeCoN-MWCNT was 0.86 V, which is 10 mV better than that of commercial 20% Pt/C (0.85 V) catalyst. The good ORR electrocatalytic activity of the FeCoN-MWCNT catalyst can be associated with the presence of M-N*_x_* centers and N-pyridinic species in the prepared catalyst, which was confirmed by the XPS analysis.

**Table 3:** Electrochemical Results of As-Synthesized Catalysts and Comparison with Other Catalysts

catalysts	*E*_onset_ (V) (ORR)	*E*_1/2_ (V) (ORR)	*E*_OER_ (V)	Δ*E* (V)	ref
FeMnN-MWCNT	0.93	0.85	1.69	0.84	this work
FeCoN-MWCNT	0.93	0.86	1.63	0.77	this work
FeNiN-MWCNT	0.92	0.82	1.58	0.76	this work
KB/FePc	0.94	0.76			(65)
TiCDC/CNT(1:3)/FePc	0.93	0.77			(65)
FeNi-COP-800	0.95	0.80			(44)
CMP-CoFe/C	0.947	0.83			(66)
FeCo-Co_4_N/N-C			1.51^a^		(67)
SWCNT + NiPc			1.6^a^		(68)

a Measured in 1 M KOH solution.

The number of electrons involved in the ORR was calculated by the K–L equation from the slopes of the K–L plots (Figure S6) and is presented in Figure 4b. All of the catalysts mainly follow a four-electron pathway for the ORR. In previous work from our workgroup using iron phthalocyanines to modify different carbon-based materials, *E*_onset_ of 0.94 V and *E*_1/2_ of 0.76 V were achieved on pyrolyzed KB/FePc electrocatalyst, and for TiCDC/CNT(1:3)/FePc, the onset potential and half-wave potential were calculated to be 0.93 and 0.77 V, respectively.^65^ Herein, FeCoN-MWCNT and FeMnN-MWCNT showed better ORR activity than KB/FePc and TiCDC/CNT(1:3)/FePc. Xiang and co-workers have reported a bimetal-phthalocyanine-based oxygen electrode (FeNi-COP-800), revealing onset and half-wave potentials of 0.95 and 0.80 V, respectively.^44^ A recent study in which a carbon framework doped with nitrogen and transition metal (CMP-CoFe/C) was synthesized by an in situ coupling strategy using metal phthalocyanines displayed *E*_onset_ of 0.947 V and *E*_1/2_ of 0.83 V.^66^ It is worth noting that in both papers,^44,66^ the preparation of catalysts was quite complex and time-consuming, but here we are able to produce similar results by only applying one-step pyrolysis to produce bimetallic and nitrogen-doped MWCNT-based electrocatalysts using corresponding metal phthalocyanines.

Further, all catalysts were also studied for the OER, as shown in Figure 4c. The OER performance of the catalysts was evaluated by the required potential (*E*_OER_) for the oxidation of water at 10 mA cm^–2^, which is commonly used in the literature about OER studies. The FeNiN-MWCNT was found to be the most active catalyst for the OER as it requires only 1.58 V to generate 10 mA cm^–2^. The difference between *E*_1/2_ and *E*_OER_ was calculated for the overall oxygen electrode activity (Table 3). FeNiN-MWCNT showed a Δ*E* value of 0.76 V in comparison with Δ*E* values of 0.84 and 0.77 V for FeMnN-MWCNT and FeCoN-MWCNT catalysts, respectively. It is known that the smaller the Δ*E* value, the closer the catalyst is to the ideal oxygen electrode. It can be seen from Figure 4c and Table 3 that FeNiN-MWCNT has the highest OER activity. Further, OER results for RuO_2_ were obtained for comparison and *E*_OER_ for RuO_2_ was found to be 1.61 V. To obtain more insight into the OER activity of the prepared catalysts, Tafel plots with the corresponding Tafel slope values are presented in Figure S7. The smallest Tafel slope was found for FeNiN-MWCNT at about 49.5 mV dec^–1^ in 0.1 M KOH followed by FeCoN-MWCNT and FeMnN-MWCNT. These Tafel slope values suggest that FeNiN-MWCNT is the best catalyst compared to other prepared materials.

In comparison to single metal phthalocyanine-modified MWCNT catalysts (FeN-MWCNT, MnN-MWCNT, CoN-MWCNT, and NiN-MWCNT, see Figure S8 andTable S3), mixed metal phthalocyanine-based MWCNT catalysts showed better bifunctional activity for the ORR and OER (especially in the case of FeCoN-MWCNT or FeNiN-MWCNT).

Next, the OER activity of the prepared catalysts compared with the literature along with the studies in which single or bimetal-based catalysts synthesized using metal phthalocyanines have been applied as OER catalysts. For example, a current density of 10 mA cm^–2^ at 1.51 V with FeCo-Co_4_N/N-C has been obtained, which is lower than the one obtained with FeNiN-MWCNT herein, but it should be noted that the measurements were conducted in 1 M KOH instead of 0.1 M KOH as used herein.^67^ Also, very recently, better OER activity on single-walled carbon nanotubes (SWCNTs) noncovalently functionalized with single MPc (e.g., NiPc, MnPc, FePc, CoPc) in terms of achieving 10 mA cm^–2^ was obtained in 1 M KOH than 0.1 M KOH.^68^ But even the best *E*_OER_ result obtained with SWCNT + NiPc (1.6 V, obtained in 1 M KOH)^68^ is 20 mV more than the *E*_OER_ of the as-synthesized FeNiN-MWCNT studied in 0.1 M KOH herein. Thus, since FeNiN-MWCNT and FeCoN-MWCNT exhibited good OER activity among the prepared electrocatalysts, it suggests that Fe and Ni or Co metal-N_4_ macrocycles together could strongly enhance the OER kinetics. Also, considering the fact that FeNiN-MWCNT and FeCoN-MWCNT showed the lowest value of Δ*E*, these materials (especially FeNiN-MWCNT) could be considered as good bifunctional electrocatalysts for both the ORR and OER.

### 3.3 Stability of the Catalysts

**Figure 5:**
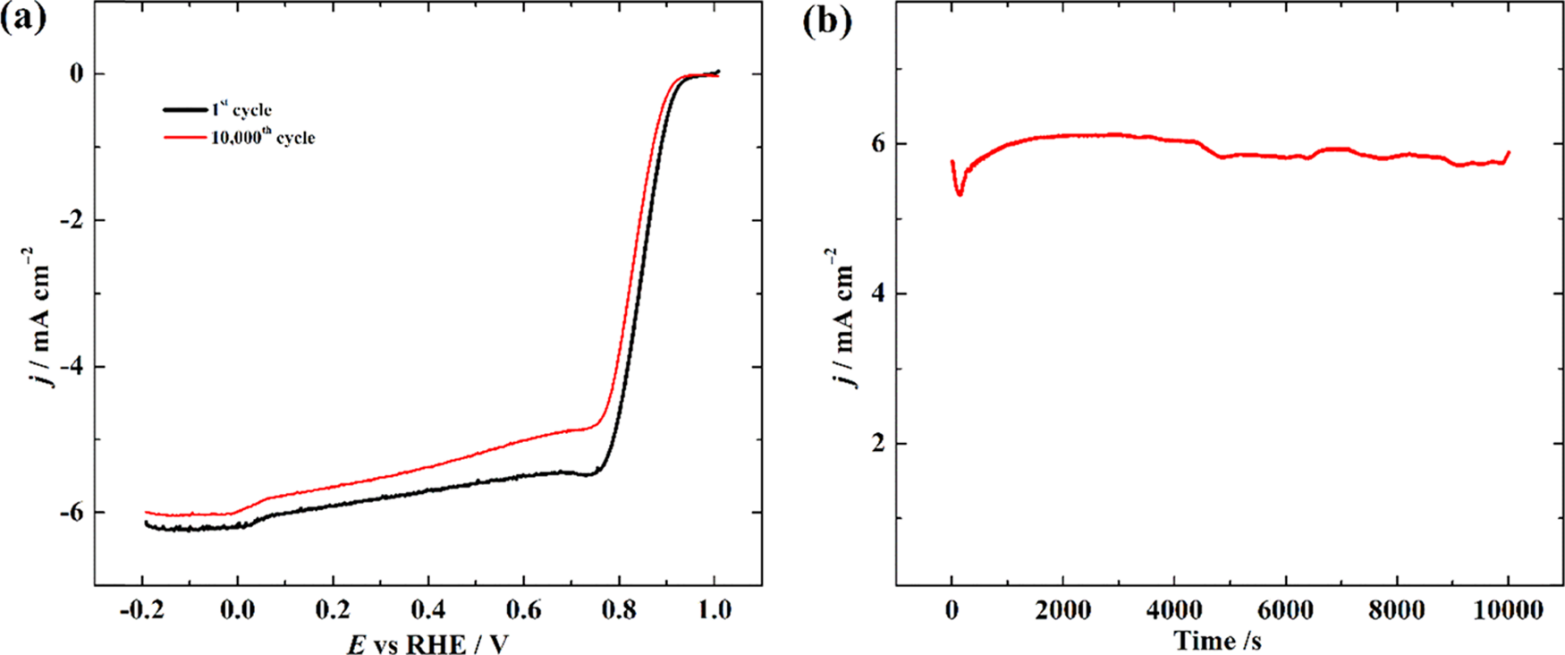
Stability testing of (a) FeCoN-MWCNT and (b) FeNiN-MWCNT catalysts in O_2_-saturated 0.1 M KOH electrolyte.

Stability tests were carried out to evaluate the catalytic stability of FeCoN-MWCNT and FeMnN-MWCNT for the ORR and FeNiN-MWCNT and FeCoN-MWCNT for the OER. As shown in Figures 5a and S9a, after 10 000 potential cycles in O_2_-saturated 0.1 M KOH solution, changes in *E*_onset_ and *E*_1/2_ for both FeCoN-MWCNT and FeMnN-MWCNT were 10 and 20 mV, respectively. In the case of OER stability test, chronoamperometric measurements were carried out at 1.6 V in 0.1 M KOH.

It was found that FeNiN-MWCNT retained 82% (Figure 5b) and FeCoN-MWCNT retained 81% (Figure S9b) of the initial OER current densities after 10 000 s. As can be seen, both electrocatalysts, FeCoN-MWCNT and FeNiN-MWCNT, are very electrochemically active and stable for the ORR and OER, respectively.

### 3.4 AEMFC Results

**Figure 6:**
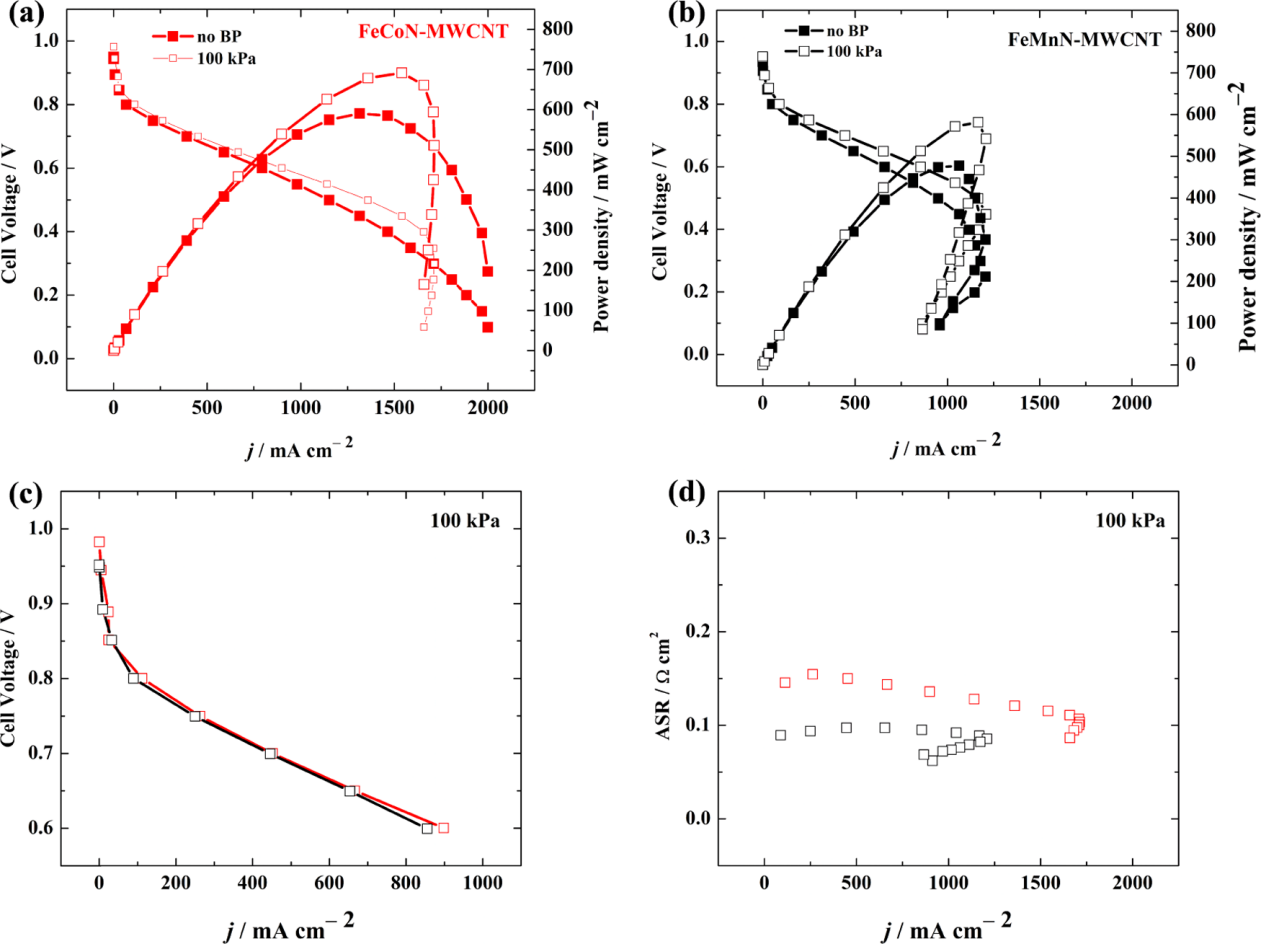
Polarization and power density curves using (a) FeCoN-MWCNT and (b) FeMnN-MWCNT cathode catalysts with cell/cathode/anode temperatures of 60/58/57 °C under H_2_/O_2_ flows of 1 slpm at atmospheric pressure and 100 kPa back-pressurization on both anode and cathode. (c) Comparison of catalytic and ohmic regions and (d) comparisons of the ASRs, of the AEMFCs under 100 kPa back-pressurization.

Given the promising ORR activities of FeCoN-MWCNT and FeMnN-MWCNT, these catalysts were tested as cathodes in H_2_-AEMFCs under similar conditions in both oxygen (Figure 6) and air (Figure S10). A comparison of the H_2_–O_2_ polarization and power density curves of the AEMFCs are shown in Figure 6a,b. At a cell temperature of 60 °C, the AEMFC based on FeCoN-MWCNT catalyst performed the best under back-pressurization, arriving at a peak power density (*P*_max_) of 692 mW cm^–2^, a current density of 898 mA cm^–2^ measured at 0.6 V, and an open-circuit voltage (OCV) of 0.98 V. The FeMnN-MWCNT-based AEMFC under the same conditions reached a *P*_max_ of 582 mW cm^–2^, a current density of 855 mA cm^–2^ measured at 0.6 V, and an OCV of 0.95 V. These results are consistent with the ORR polarization curves recorded in O_2_-saturated 0.1 M KOH shown in Figure 4. To our knowledge, these power and current density values are among the highest in the literature using MN_4_ macrocycle-derived electrocatalysts at the cathode (Table S4). For instance, Wang et al. used a highly active ORR catalyst, FeCoPc/C,^51^ which exhibited an onset potential as high as 1.02 V combined with a highly conductive state-of-the-art LDPE15-AEM, ETFE ionomer, and PtRu anode in an AEMFC tested at 80 °C. Owing to the higher cell temperature and superior materials used in their study, they reported a remarkable *P*_max_ value of around 1260 mW cm^–2^. A lower value (*P*_max_ = 473 mW cm^–2^)^28^ was observed at a comparable temperature of 60 °C using SiCDC/CNT/CoPc by Praats et al. Both fuel cells exhibited similar performance in the catalytic and ohmic regions of the AEMFC, as shown in Figure 6c, with only a slight difference in current densities at 0.6 V in favor of the FeCoN-MWCNT. Figure 6d shows that the area specific resistance (ASR) in the FeMnN-MWCNT cell was slightly lower than in the FeCoN-MWCNT cell, indicating that the membranes were adequately hydrated, therefore enabling efficient hydroxide conduction and water back-diffusion.^69^ The mass transport regions of the FeMnN-MWCNT AEMFC in both cases of atmospheric pressure and 100 kPa did not extend to high current densities beyond 1250 mA cm^–2^, as did the FeMnN-MWCNT AEMFC. We theorize that further optimization of the CCL could help to enhance the ability to reduce flooding and retain back-diffused water to participate in the ORR process as supported by Gutru et al.^70^

After the acquisition of the H_2_–O_2_ polarization curves, the oxidant was switched to (CO_2_-free) air, while all of the other fuel cell conditions were maintained. The H_2_-air polarization curves shown in Figure S10 exhibit a similar trend, wherein FeCoN-MWCNT AEMFC performed better than FeMnN-MWCNT AEMFC in terms of *P*_max_ and current density at 0.6 V.

### 3.5 AEMEL Results

To test the performance of the FeNiN-MWCNT and RuO_2_ cells, linear sweep voltammetry was conducted. The cells show similar response to the RDE linear sweep voltammetry (Figure 4c) in that FeNiN-MWCNT showed slightly higher current than the RuO_2_ at similar catalyst loading (Figure S11a). Chronoamperometry was conducted using both FeNiN-MWCNT and RuO_2_ cells at 1.8 V. The cell based on RuO_2_ began with current around 110 mA cm^–2^, but the current increased and stabilized around 140 mA cm^–2^, possibly due to membrane break-in (Figure S11b). The observed current density of the FeNiN-MWCNT cell showed an initial current around 120 mA cm^–2^, which decayed over 2 h to ∼70 mA cm^–2^ (Figure S11b), possibly due to oxidative degradation of the FeNiN catalyst or the MWCNT substrate. In comparison with other state-of-the-art AEMEL systems, the benchmark material RuO_2_ used herein has slightly lower current density than expected, for example, IrO_2_ has an observed current density of 399 mA cm^–2^ under comparable conditions (Table S5). The lower current density is a result of unoptimized AEMEL conditions such as ionomer:catalyst ratio, membrane thickness, and GDL electrical contact/gas removal efficiency. Nonetheless, FeNiN-MWCNT compares well with the state-of-the-art RuO_2_ benchmarking conducted herein, and its performance can be improved by further optimization.

## 4 Conclusions

In conclusion, we presented a one-step pyrolysis method for the synthesis of mixed transition-metal M-N-C-type catalysts, in which iron phthalocyanine was used in combination with different metal phthalocyanines (e.g., FeNi; FeMn, and FeCo) and was studied for the oxygen electrocatalytic reactions (ORR and OER) in alkaline electrolyte. The presence of catalytic M-N*_x_* center enhances the oxygen reduction reaction as well as the oxygen evolution reaction. The presence of metal-nitrogen species was confirmed in all catalyst materials by XPS. FeCoN-MWCNT was found to be the most active catalyst for the ORR, and FeNiN-MWCNT was the most active for the OER. However, the best bifunctional properties in terms of the lowest Δ*E* value were achieved with FeCoN-MWCNT and FeNiN-MWCNT catalysts. From the electrochemical device tests, FeCoN-MWCNT catalyst exhibited a very good peak power density value (692 mW cm^–2^) in an AEMFC and FeNiN-MWCNT compares well with the state-of-the-art RuO_2_ in an AEMEL. The present study provides insights into potential ways of preparing mixed transition-metal-based MN*_x_* species bifunctional catalysts with high electrocatalytic activity.

## Supporting Information Available

Results of physical characterization (SEM, EDX, and XPS), some electrochemical characterization results of the catalysts (CV, RDE), and tables comparing AEMFC and AEMEL results (PDF)
