# Supplementary material for: Bifunctional Oxygen Electrocatalysis on Mixed Metal Phthalocyanine-Modified Carbon Nanotubes Prepared via Pyrolysis
Source: ACS Appl Mater Interfaces. 2021 Aug 24;13(35):41507–16. doi: 10.1021/acsami.1c06737 (PMC8589254; doi:10.1021/acsami.1c06737)
Supplement: Supplementary file 1 — am1c06737_si_001.pdf [file am1c06737_si_001.pdf]

Supporting Information

Bifunctional Oxygen Electrocatalysis on Mixed Metal Phthalocyanine-Modified Carbon Nanotubes Prepared via Pyrolysis

Yogesh Kumar,[†] Elo Kibena-Põldsepp,[†] Jekaterina Kozlova,[‡] Mihkel Rähn,[‡] Alexey Treshchalov,[‡] Arvo Kikas,[‡] Vambola Kisand,[‡] Jaan Aruväli,[§] Aile Tamm,[‡] John C. Douglin,[∥] Scott J. Folkman,[x] Ilario Gelmetti,[x] Felipe A. Garcés-Pineda,[x] José Ramón Galán-Mascarós,[x,z] Dario R. Dekel,[∥,⊥,*] and Kaido Tammeveski[†,*]

[†]Institute of Chemistry, University of Tartu, Ravila 14a, 50411 Tartu, Estonia

[‡]Institute of Physics, University of Tartu, W. Ostwald Str. 1, 50411 Tartu, Estonia

[§]Institute of Ecology and Earth Sciences, University of Tartu, Vanemuise 46, 51014 Tartu, Estonia

[∥]The Wolfson Department of Chemical Engineering, Technion – Israel Institute of Technology, 3200003 Haifa, Israel

[x]Institute of Chemical Research of Catalonia (ICIQ), The Barcelona Institute of Science and Technology (BIST), 43007 Tarragona, Spain

[z]Catalan Institution for Research and Advanced Studies (ICREA), Passeig Llüis Companys 23, 08010 Barcelona, Spain

[⊥]The Nancy & Stephen Grand Technion Energy Program (GTEP), Technion – Israel Institute of Technology, 3200003, Haifa, Israel

Table S1. Elemental composition of as-synthesized catalysts determined by SEM-EDX (wt%).

Element	FeMnN-MWCNT	FeCoN-MWCNT	FeNiN-MWCNT
**C**	82.6	81.7	83.6
**N**	5.9	8.5	6.5
**O**	6.9	5.0	4.7
**Fe**	1.9	2.5	2.9
**Mn / Co / Fe**	2.4	2.1	2.3

[*]Corresponding author. Phone: +372 7375168. Email: kaido.tammeveski@ut.ee (K. Tammeveski).
*Corresponding author. Phone: +972 77 8871792. Email: dario@technion.ac.il (D. Dekel).

Table S2. Surface content of nitrogen species for catalysts determined by XPS analysis (at%).

N species	FeMnN-MWCNT	FeCoN-MWCNT	FeNiN-MWCNT
**N-pyridinic**	2.2	3.0	1.8
**M-N_x**	0.9	0.8	0.5
**N-pyrrolic**	1.0	1.3	0.9
**N-graphitic**	0.4	0.5	0.3
**N-O**	0.3	0.2	-
**bulk N-H**	0.2	0.3	-

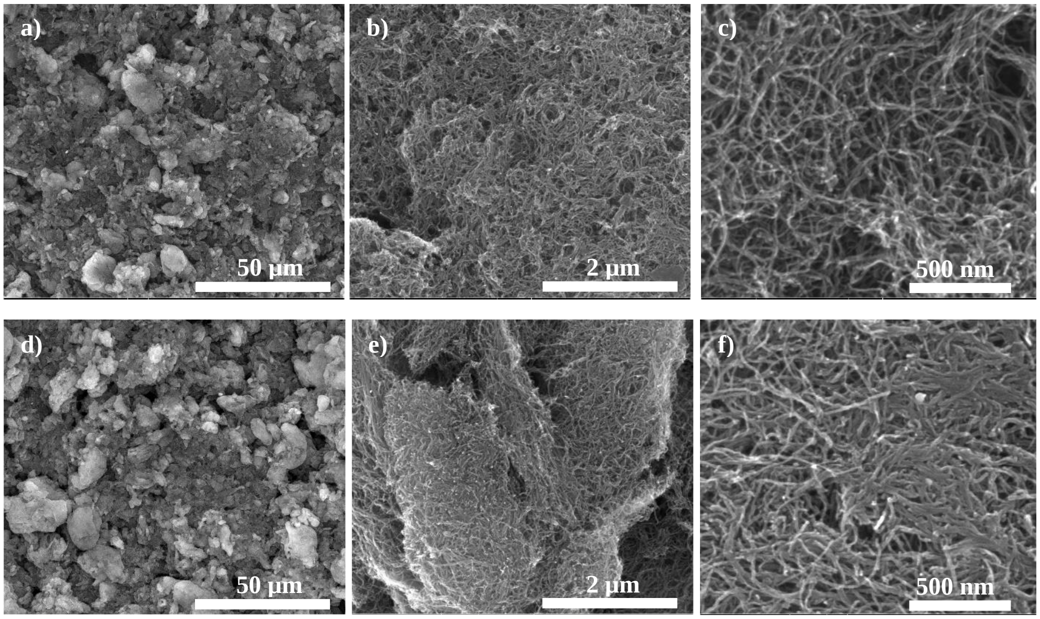

Fig. S1. SEM images of (a-c) FeCoN-MWCNT and (d-f) FeNiN-MWCNT.

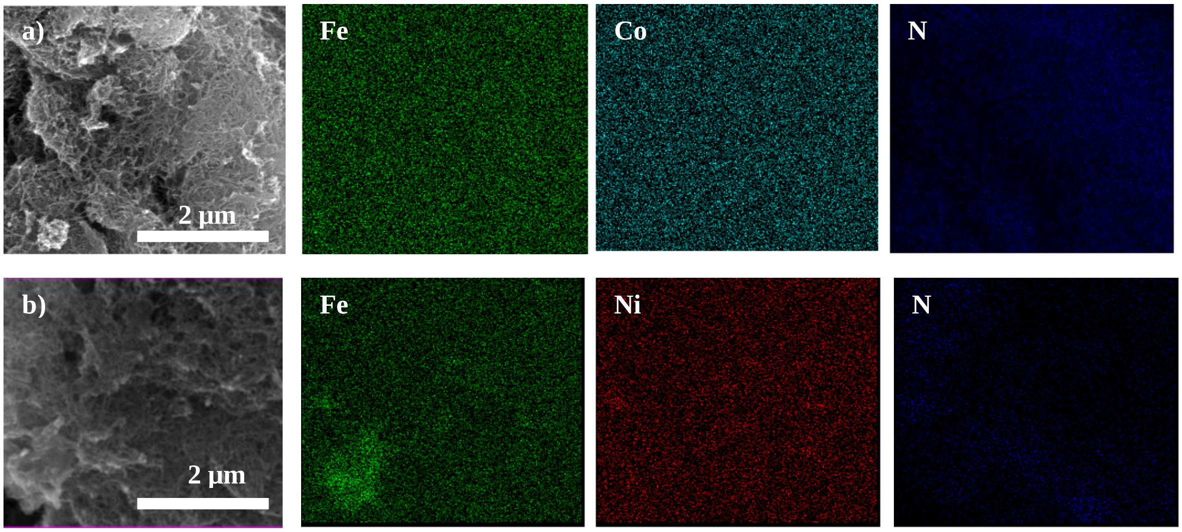

Fig S2. SEM-EDX analysis with elemental mapping of (a) FeCoN-MWCNT and (b) FeNiN-MWCNT.

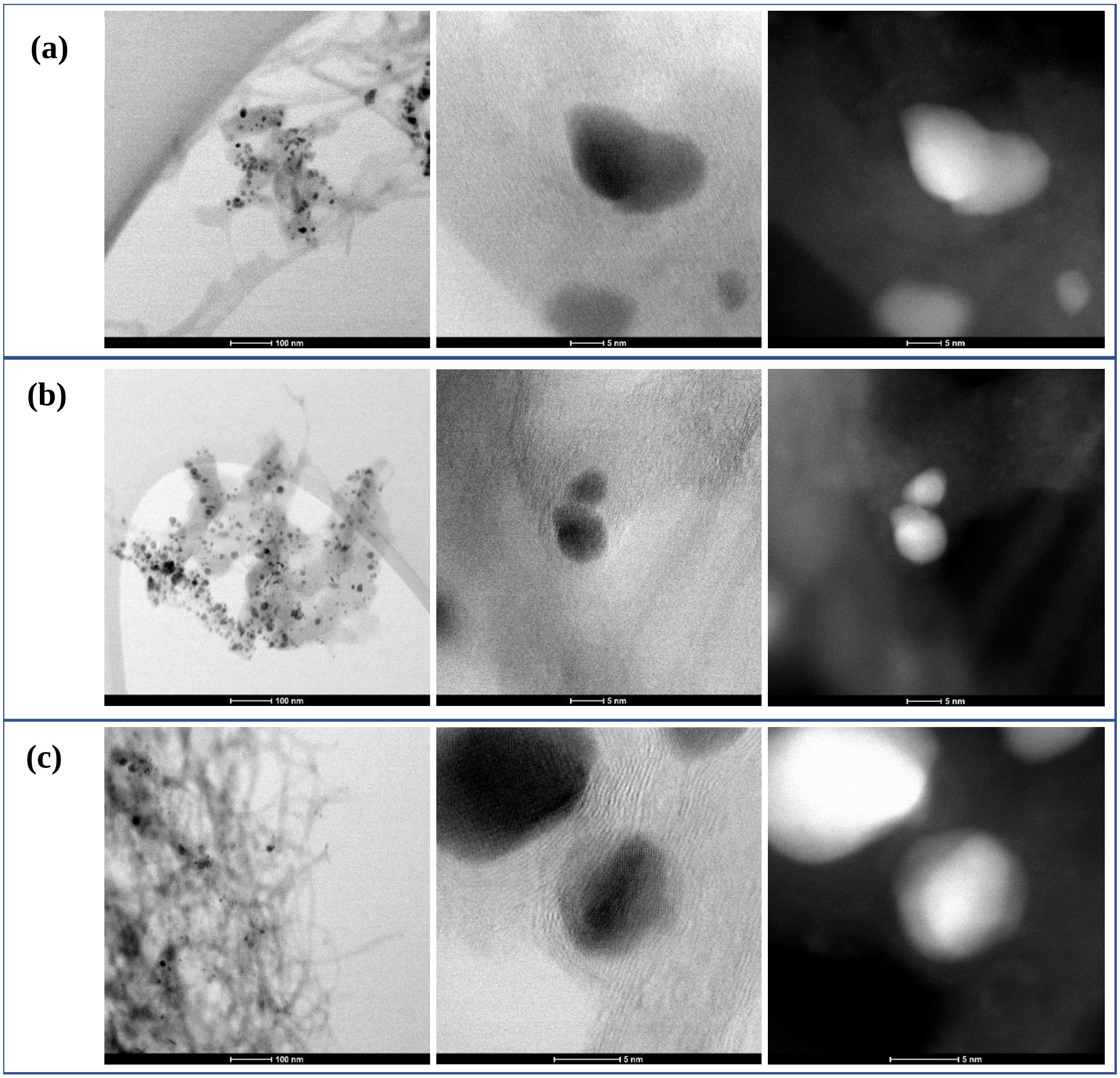

Fig. S3. Bright field and high-angle annular dark field (HAADF) images of (a) FeMnN-MWCNT, (b) FeCoN-MWCNT, and (c) FeNiN-MWCNT.

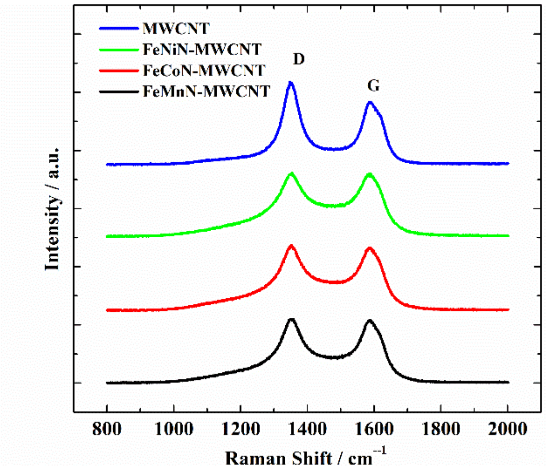

Fig. S4. Raman spectra of pristine and as-synthesized MWCNT-based catalysts.

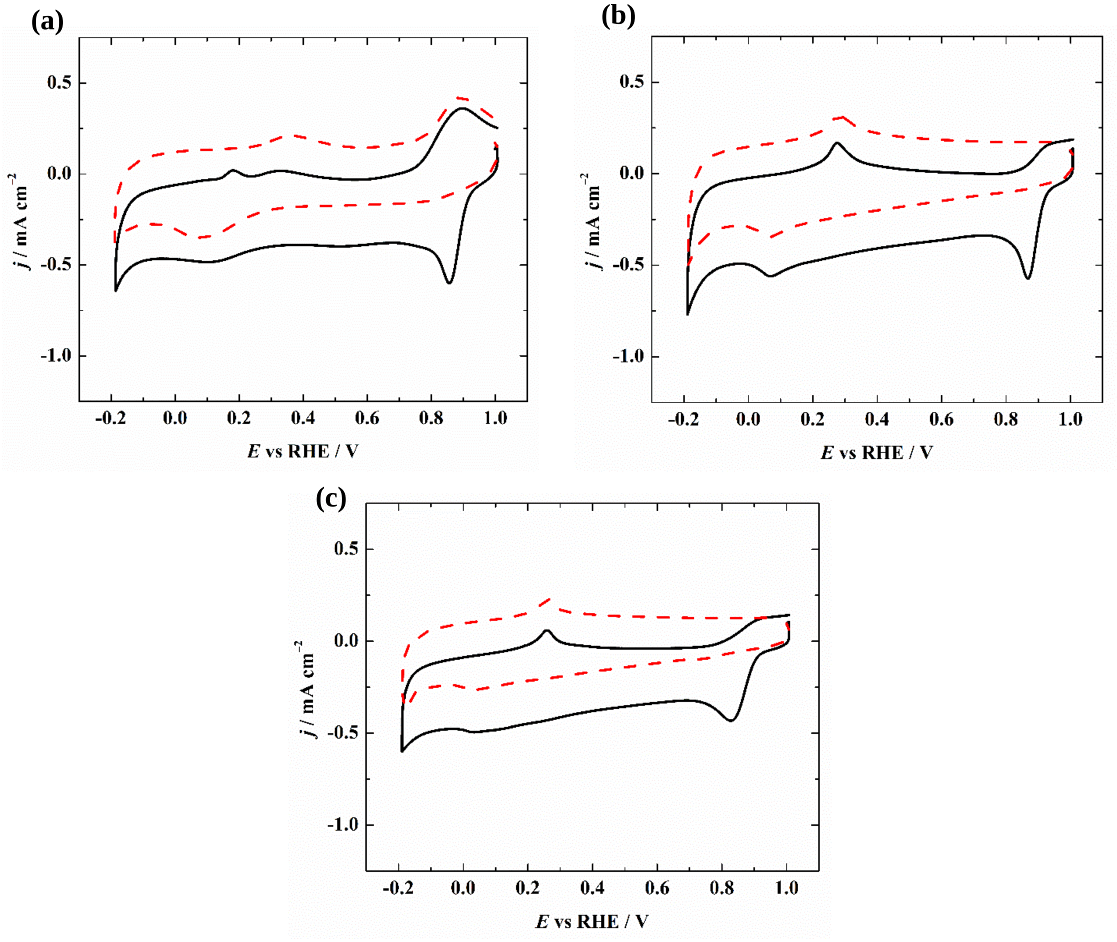

Fig. S5. Cyclic voltammograms of (a) FeMnN-MWCNT, (b) FeCoN-MWCNT and (c) FeNiN-MWCNT in Ar-saturated (red dashed line) and O_2-saturated (black solid line) 0.1 M KOH electrolyte at 10 mV s^{-1}.

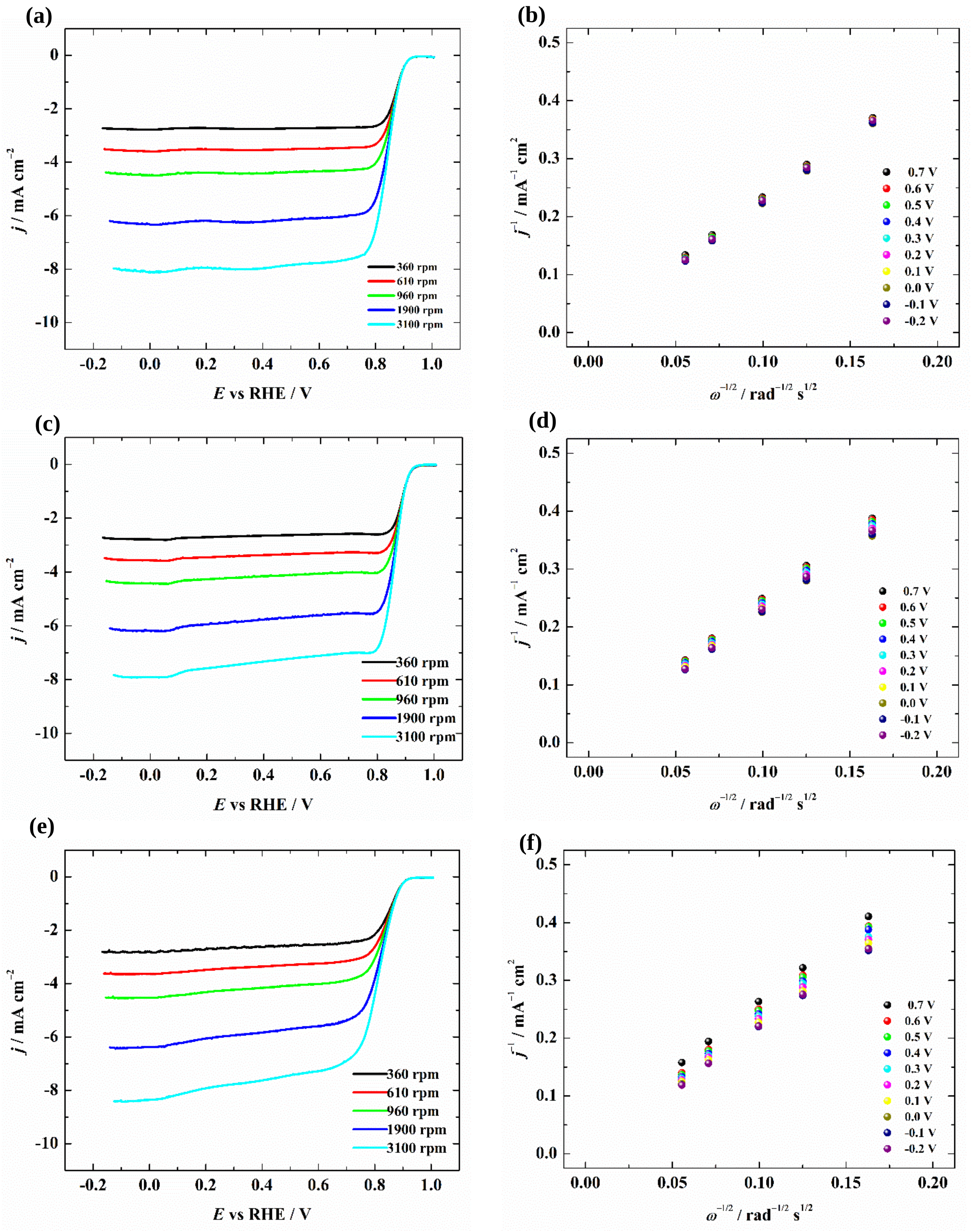

Fig. S6. ORR polarization curves for: (a) FeMnN-MWCNT, (c) FeCoN-MWCNT and (e) FeNiN-MWCNT at different rotation rates in O_2-saturated 0.1 M KOH electrolyte (ν = 10 mV s^{-1}). Koutecky-Levich plots for the ORR derived from the RDE data for (b) FeMnN-MWCNT, (d) FeCoN-MWCNT and (f) FeNiN-MWCNT.

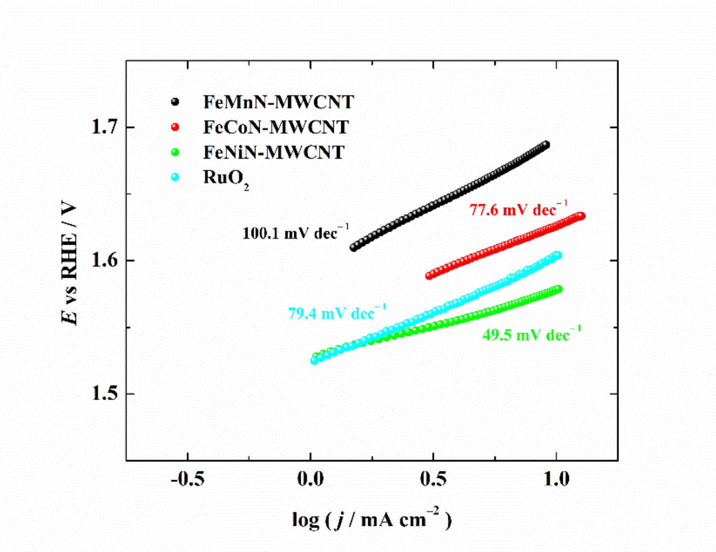

Fig. S7. Tafel plots for OER on FeMnN-MWCNT, FeCoN-MWCNT and FeNiN-MWCNT in Ar-saturated 0.1 M KOH electrolyte.

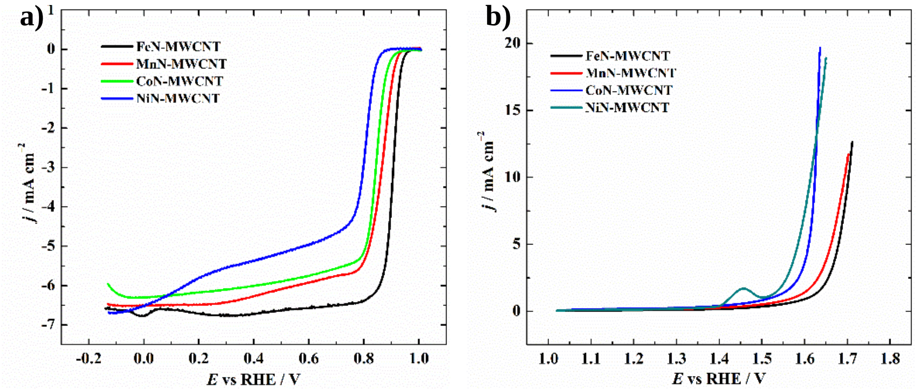

Fig. S8. (a) ORR and (b) OER polarization curves of single metal phthalocyanine-modified MWCNT catalysts in (a) O_2-saturated and (b) Ar-saturated 0.1 M KOH electrolyte at 1900 rpm, v= 10 mV s^{-1}.

Table S3. Electrochemical results of single metal phthalocyanine modified MWCNT.

Catalysts	E_{onset}/V	$E_{1/2}$/V	E_{OER}/V	ΔE/V
**FeN-MWCNT**	0.95	0.90	1.70	0.80
**MnN-MWCNT**	0.93	0.86	1.69	0.83
**CoN-MWCNT**	0.92	0.83	1.62	0.79
**NiN-MWCNT**	0.86	0.79	1.61	0.82

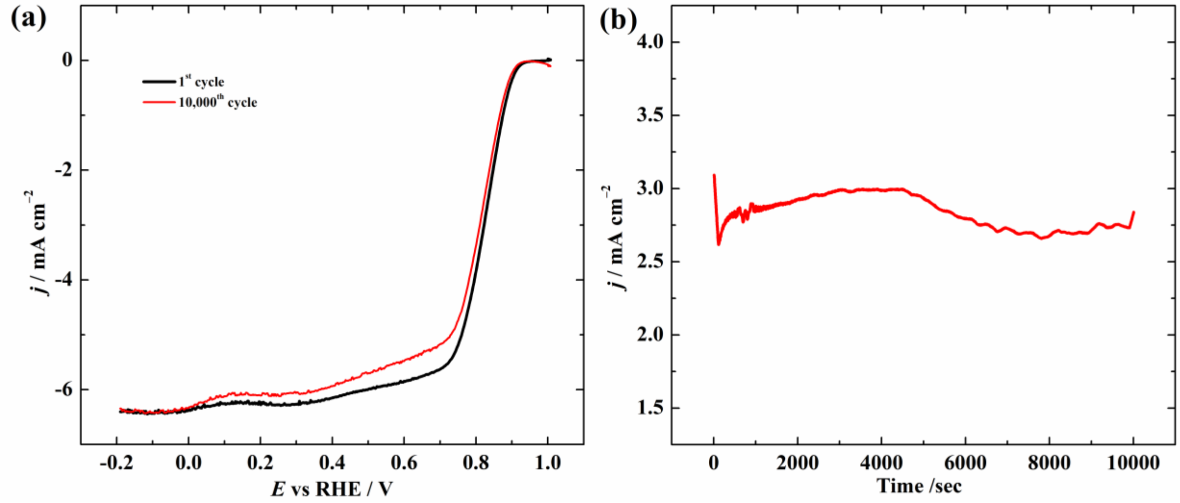

Fig. S9. Stability testing of (a) FeMnN-MWCNT and (b) FeCoN-MWCNT catalysts in O_2-saturated 0.1 M KOH electrolyte.

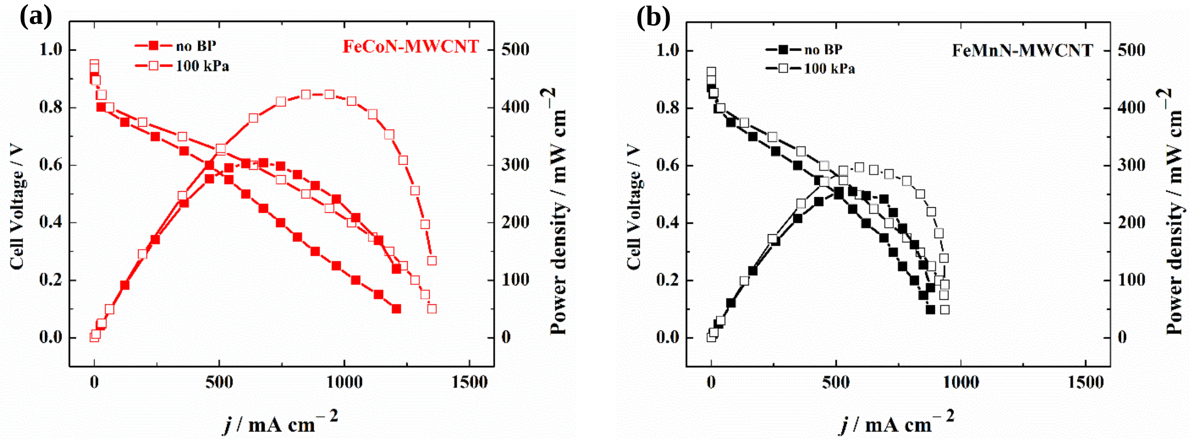

Fig. S10. Polarization and power density curves using (a) FeCoN-MWCNT and (b) FeMnN-MWCNT cathode catalyst with fuel cell/cathode/anode temperatures of 60/58/57 °C under H_2/(CO_2-free) air flows of 1 slpm at atmospheric pressure and 100 kPa back-pressurization on both anode and cathode.

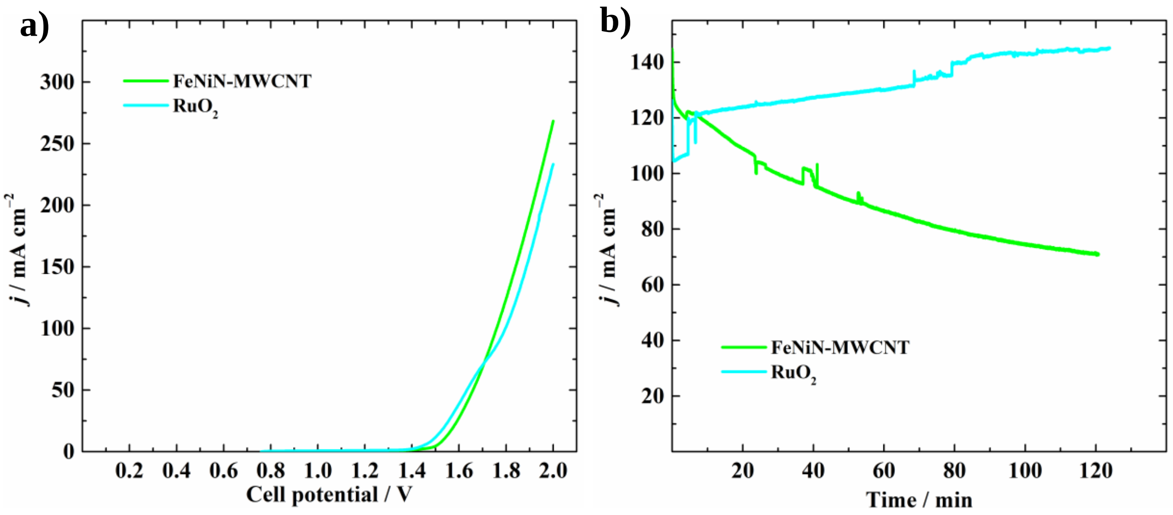

Fig. S11. (a) Linear sweep voltammetry and (b) chronoamperometry of FeNiN-MWCNT and RuO_2 of MEAs conducting on VM-FAA-3-10-rf anion exchange membrane, Pt/C cathode, in 0.1 M KOH at 60 °C.

Table S4. Comparison of H_2-O_2 AEMFC performance using MN_4 macrocycle derived electrocatalysts at the cathode.

Catalyst	Cathode loading (mg cm^{-2})	Anode loading (mg cm^{-2})	T (ºC)	Membrane	Ionomer (ionomer-to-carbon ratio, I/C)	Anode/Cathode gas flow rates (mL min^{-1})	Anode/Cathode backpressures (barg)	P_{max} (mW cm^{-2})	P_{max} for Pt/C (mW cm^{-2})	*j* at 0.6 V (mA cm^{-2})	*j* at 0.6 V for Pt/C (mA cm^{-2})	OCV (V)	Ref.
FeCoN-MWCNT<br>FeMnN-MWCNT	0.745	0.729<br>(40% Pt/C)	60	FAA-3-5	Fumatech<br>(I/C=3:7)	1000/1000	1/1	692<br>582	-	898<br>855	-	0.98<br>0.95	This work
MPc/C<br>(M=Fe,Co,Ni,Mn)<br>C= Carbon Black	2	-<br>(50% Pt/C)	50	Tokuyama A901	Tokuyama AS-4<br>(I/C= 1:4)	200/200	0.4/0.4	85* (Fe)<br>87* (Ni)<br>96* (Mn)<br>105* (Co)	-	90-160*	80-175*	0.84* (Fe)<br>0.90* (Ni)<br>0.88* (Mn)<br>0.88* (Co)	1
MPc/C<br>(M=Co,Cu,Zn,Ni)<br>C= Carbon Black	3	0.5<br>(40% Pt/C)	RT	Tokuyama A901	Nafion<br>(I/C =1:3)	70/100	0/0	12.6 (Co)<br>6.0 (Ni)<br>6.8 (Cu)<br>6.1 (Zn)	37.7	6-20*	60*	0.90 (Co)<br>0.73 (Ni)<br>0.87 (Cu)<br>0.73 (Zn)	2
FePc/C	1	0.4 mg_{Pt} cm^{-2}	55	Tokuyama A901	Tokuyama AS-4<br>(I/C = 0.6)	200/100	0/0	120	310*	190*	400*	0.93	3
MPc/MWCNT<br>(M=Co,Fe)	0.6	0.4<br>(46% Pt/C)	45	Tokuyama A201	Tokuyama AS-4	400/200	0/0	60 (Fe)<br>100 (Co)	120	70* (Fe)<br>146* (Co)	195*	0.96* (Fe)<br>0.96* (Co)	4
$CoFeN_x$/C<br>C= Carbon Black	2.5	0.4<br>(40% Pt/C)	60	Tokuyama A201	Tokuyama AS-4<br>(20 wt%)	200/200	0/0	37	170	57	260	1.00	5
Cu SAC	2	0.5 mg_{Pt} cm^{-2}	70	-	-	-	-	196	236	230	320	0.85	6
SiCDC/CNT(1:3)/<br>CoPc	0.7	0.7 mg cm^{-2}<br>(40% PtRu/C)	60	ETFE-based AEM	-<br>(I/C = 3:7)	1000/1000	0/0	473	-	637	-	0.92	7
Pyrolysed KB/FePc	2.0	0.8 mg cm^{-2}<br>(PtRu/C)	60	HMT-PMBI polymer	HMT-PMBI	1000/1000	2/2	186	350*	220	570	1.00	8
Fe-N_x-CNT/PC	2	0.5 mg_{Pt} cm^{-2}	80	-	-<br>(I/C = 1:3)	1200/400	0/0	380	540	498	800	0.98	9
FeCoPc/C	0.30	0.7 mg_{PtRu} cm^{-2}	80	LDPE	ETFE<br>(I/C = 1:4)	1000/1000	0/0	1260	2000	1700	3200	0.88	10

*Data is estimated from Figure

Table S5. Comparison of AEMEL based on FeNiN-MWCNT and RuO_2 used herein with other benchmark systems.

Membrane Electrode Assembly								Operating Conditions				Ref.
Anode			Cathode			Membrane	Ionomer					
GDL	Catalyst	Loading (mg cm^{-2})	GDL	Catalyst	Loading (mg/cm^{-2})			Electrolyte	T (°C)	Applied Voltage (V)	Current Density (mA cm^{-2})	
Ti Mesh	FeNiN-MWNT	1.9	Carbon paper	Pt black	0.5	VM-FAA-3-10-rf	FAA-3	0.1 M KOH	60	1.8	70	This Work
Ti Mesh	RuO2	1.8	Carbon paper	Pt black	0.5	VM-FAA-3-10-rf	FAA-3	0.1 M KOH	60	1.8	140	This Work
Ti Foam	IrO_2	2.9	Ti Foam	Pt Black	3.2	A-201, Tokuyama	AS-4	Distilled water	50	1.8	399	11
Ni foam	Ni-Fe	40	Stainless steel	Ni-Mo	40	(xQAPS)	(xQAPS)	Ultrapure water	70	1.85	400	12
Ni foam	Ni/CeO_2-La_2O_3)/C	40	Carbon cloth	$CuCoO_3$	40	Mg-Al LDH	PTFE	0.1M KOH	70	2.2	208	13
Ni foam	Ni foam	-	Ni foam	$NiCo_2O_4$	10	-	qPPO	10% KOH	50	1.85	135	14
Ni foam	$Cu_{0.81}Co_{2.19}O_4$NS	4	Ni foam	Co_3S_4	3	X37-50	-	1M KOH	45	2.0	431	15
Platinized Ti	Ni_2Fe_1	3	SGL 29 BCE	Ni_9Mo_1/C	2	HTMA-DAPP	TMA	1M NaOH	60	1.8	906	16

References

(1) Guo, J.; He, H.; Chu, D.; Chen, R. OH^--Binding Effects on Metallophthalocyanine Catalysts for O2 Reduction Reaction in Anion Exchange Membrane Fuel Cells. *Electrocatalysis* **2012**, *3*, 252–264. https://doi.org/10.1007/s12678-012-0106-1.

(2) Zhu, T.; Qing, X.; Xu, P.; Song, Y.; Qiao, J. H_2/O_2 Alkaline Membrane Fuel Cell Performances Using Carbon-Supported Metal Phthalocyanine (MPc/C,M = Co, Cu, Zn, Ni) as Cathode Catalysts. *ECS Trans.* **2015**, *66*, 105–110. https://doi.org/10.1149/06603.0105ecst.

(3) Miller, H. A.; Bellini, M.; Oberhauser, W.; Deng, X.; Chen, H.; He, Q.; Passaponti, M.; Innocenti, M.; Yang, R.; Sun, F.; Jiang, Z.; Vizza, F. Heat Treated Carbon Supported Iron(II)Phthalocyanine Oxygen Reduction Catalysts: Elucidation of the Structure-Activity Relationship Using X-Ray Absorption Spectroscopy. *Phys. Chem. Chem. Phys.* **2016**, *18*, 33142–33151. https://doi.org/10.1039/c6cp06798k.

(4) Kruusenberg, I.; Matisen, L.; Shah, Q.; Kannan, A. M.; Tammeveski, K. Non-Platinum Cathode Catalysts for Alkaline Membrane Fuel Cells. *Int. J. Hydrogen Energy* **2012**, *37*, 4406–4412. https://doi.org/10.1016/j.ijhydene.2011.11.143.

(5) Jiang, R.; Chu, D. Comparative Study of $CoFeN_x/C$ Catalyst Obtained by Pyrolysis of Hemin and Cobalt Porphyrin for Catalytic Oxygen Reduction in Alkaline and Acidic Electrolytes. *J. Power Sources* **2014**, *245*, 352–361. https://doi.org/10.1016/j.jpowsour.2013.06.123.

(6) Cui, L.; Cui, L.; Li, Z.; Zhang, J.; Wang, H.; Lu, S.; Xiang, Y. A Copper Single-Atom Catalyst towards Efficient and Durable Oxygen Reduction for Fuel Cells. *J. Mater. Chem. A* **2019**, *7*, 16690–16695. https://doi.org/10.1039/c9ta03518d.

(7) Praats, R.; Käärik, M.; Kikas, A.; Kisand, V.; Aruväli, J.; Paiste, P.; Merisalu, M.; Sarapuu, A.; Leis, J.; Sammelselg, V.; Douglin, J. C.; Dekel, D. R.; Tammeveski, K. Electroreduction of Oxygen on Cobalt Phthalocyanine-Modified Carbide-Derived Carbon/Carbon Nanotube Composite Catalysts. *J. Solid State Electrochem.* **2021**, *25*, 57–71. https://doi.org/10.1007/s10008-020-04543-z.

(8) Praats, R.; Käärik, M.; Kikas, A.; Kisand, V.; Aruväli, J.; Paiste, P.; Merisalu, M.; Leis, J.; Sammelselg, V.; Zagal, J. H.; Holdcroft, S.; Nakashima, N.; Tammeveski, K. Electrocatalytic Oxygen Reduction Reaction on Iron Phthalocyanine-Modified Carbide-Derived Carbon/Carbon Nanotube Composite Electrocatalysts. *Electrochim. Acta* **2020**, *334*, 135575. https://doi.org/10.1016/j.electacta.2019.135575.

(9) Sa, Y. J.; Seo, D. J.; Woo, J.; Lim, J. T.; Cheon, J. Y.; Yang, S. Y.; Lee, J. M.; Kang, D.; Shin, T. J.; Shin, H. S.; Jeong, H. Y.; Kim, C. S.; Kim, M. G.; Kim, T. Y.; Joo, S. H. A General Approach to Preferential Formation of Active Fe-Nx Sites in Fe-N/C Electrocatalysts for Efficient Oxygen Reduction Reaction. *J. Am. Chem. Soc.* **2016**, *138*, 15046–15056. https://doi.org/10.1021/jacs.6b09470.

(10) Wang, L.; Bellini, M.; Miller, H. A.; Varcoe, J. R. A High Conductivity Ultrathin Anion-Exchange Membrane with 500+ h Alkali Stability for Use in Alkaline Membrane Fuel Cells That Can Achieve 2 W cm^{-2} at 80 °C. *J. Mater. Chem. A* **2018**, *6*, 15404–15412. https://doi.org/10.1039/c8ta04783a.

(11) Leng, Y.; Chen, G.; Mendoza, A. J.; Tighe, T. B.; Hickner, M. A.; Wang, C. Y. Solid-State Water Electrolysis with an Alkaline Membrane. *J. Am. Chem. Soc.* **2012**, *134*,

9054–9057. https://doi.org/10.1021/ja302439z.

(12) Xiao, L.; Zhang, S.; Pan, J.; Yang, C.; He, M.; Zhuang, L.; Lu, J. First Implementation of Alkaline Polymer Electrolyte Water Electrolysis Working Only with Pure Water. *Energy Environ. Sci.* **2012**, *5*, 7869–7871. https://doi.org/10.1039/c2ee22146b.

(13) Zeng, L.; Zhao, T. S. Integrated Inorganic Membrane Electrode Assembly with Layered Double Hydroxides as Ionic Conductors for Anion Exchange Membrane Water Electrolysis. *Nano Energy* **2015**, *11*, 110–118. https://doi.org/10.1016/j.nanoen.2014.10.019.

(14) Chanda, D.; Hnát, J.; Bystron, T.; Paidar, M.; Bouzek, K. Optimization of Synthesis of the Nickel-Cobalt Oxide Based Anode Electrocatalyst and of the Related Membrane-Electrode Assembly for Alkaline Water Electrolysis. *J. Power Sources* **2017**, *347*, 247–258. https://doi.org/10.1016/j.jpowsour.2017.02.057.

(15) Park, Y. S.; Lee, J. H.; Jang, M. J.; Jeong, J.; Park, S. M.; Choi, W. S.; Kim, Y.; Yang, J.; Choi, S. M. Co3S4 Nanosheets on Ni Foam via Electrodeposition with Sulfurization as Highly Active Electrocatalysts for Anion Exchange Membrane Electrolyzer. *Int. J. Hydrogen Energy* **2020**, *45*, 36–45. https://doi.org/10.1016/j.ijhydene.2019.10.169.

(16) Li, D.; Park, E. J.; Zhu, W.; Shi, Q.; Zhou, Y.; Tian, H.; Lin, Y.; Serov, A.; Zulevi, B.; Baca, E. D.; Fujimoto, C.; Chung, H. T.; Kim, Y. S. Highly Quaternized Polystyrene Ionomers for High Performance Anion Exchange Membrane Water Electrolysers. *Nat. Energy* **2020**, *5*, 378–385. https://doi.org/10.1038/s41560-020-0577-x.
